# Comparison of young male mice of two different strains (C57BL/6J and the hybrid B6129SF1/J) in selected behavior tests: a small scale study

**DOI:** 10.1186/s42826-022-00140-5

**Published:** 2022-10-02

**Authors:** Kristine Eraker Aasland Hansen, Alexandra M. Hudecová, Fred Haugen, Eystein Skjerve, Erik Ropstad, Karin E. Zimmer

**Affiliations:** 1 Section for Experimental Biomedicine, Department of Production Animal Clinical Sciences, Norwegian University of Life Sciences, Ås, Norway; 2 Environmental Chemistry Department, Norwegian Institute for Air Research, Ås, Norway; 3 Division of Work Physiology, National Institute of Occupational Health, Oslo, Norway; 4 Section for Animal Welfare, Department of Production Animal Clinical Sciences, Norwegian University of Life Sciences, Ås, Norway; 5 Section for Stationary Clinics, Department of Production Animal Clinical Sciences, Norwegian University of Life Sciences, Ås, Norway; 6 Physiology Unit, Department of Preclinical Sciences and Pathology, Faculty of Veterinary Medicine, Norwegian University of Life Sciences, Ås, Norway

**Keywords:** Mouse strain, C57BL/6J, Hybrid B6129SF1/J, Behavior test, Open field test, Barnes maze test, Stress test

## Abstract

**Background:**

All mouse strains are different, before choosing a strain for a large study, a small scale study should be done. In this study, we compared young males of two mouse strains, C57BL/6J and the hybrid B6129SF1/J, and gained knowledge on their performance in three different behavioral tests; open field (OF) test, Barnes maze (BM) test and a restraint stress test.

**Results:**

We found that the young males of the C57BL/6J strain spent more time moving in the OF. In the BM, the hybrid covered less ground before reaching the goal box during the first three sessions, than the C57BL/6J. The hybrid left more fecal pellets than C57BL/6J both in OF and BM. During the stress test, the C57BL/6J had a lower corticosterone response than the hybrid.

**Conclusions:**

Our findings indicate that the C57BL/6J has a presumably higher locomotor activity and/or explorative behavior than the hybrid, while the hybrid appeared more sensitive to stress.

## Background

Differences in behavior between mouse strains are frequently observed in animal facilities, and certain strains are well known, and often chosen, for a certain type of behavior. Also, in behavior tests differences between strains are well documented [1]. Thus, the strain used for behavioral tests should be carefully considered. For future behavior experiments, we wanted to test two possible strains of mice, to see which one would be more beneficial for our purposes. The ideal strain would be a good learner, robust in stressful situations, gentle mothers, easy to breed, group-house and handle. The C57BL/6 J mouse is the most widely used inbred strain in laboratory animal research and is used in a widespread of research fields, including cancer research, diabetes/obesity research and behavioral/learning research. The breeders`, Jackson Laboratory, webpage “Mouse Phenome Database” (https://phenome.jax.org/) lists 282 studies using this strain. The hybrid B6129SF1/J is described as having hybrid vigor and is used in for example tissue transplantation research. It has also been used in behavior studies for several years [2]. The database shows 9 studies using the hybrid. When asking the database to compare the two strains, there were no datasets found and several searches in several other search tools showed that research comparing these two strains in behavioral tests is scarce. In the experiment described, only the two first beneficial traits listed were tested; learning ability and stress-responsiveness. The two mouse strains were tested in three different behavioral tests, an open field (OF) test, a Barnes maze (BM) test and a restraint stress test. Previous reports conclude that C57BL/6J is a locomotory active strain in the OF while the hybrid would be in between C57BL/6J and 129 [3]. Chan et al*.* found the C57BL/6 to have a larger corticosterone output than the similar hybrid in response to acute stress [4]. However, still, little is known about differences between these two in the BM and corticosterone response to stress. It was not considered comparing the two strains to 129 as this strain has previously shown low rates of exploration and poor learning performance in BM [5]. The aim of this article was to add to the knowledge about the behavior of the C57BL/6J and the hybrid B6129SF1/J mouse strain in the OF, BM and a restraint stress test.

## Results

### Open field (OF) test

**Fig. 1:**
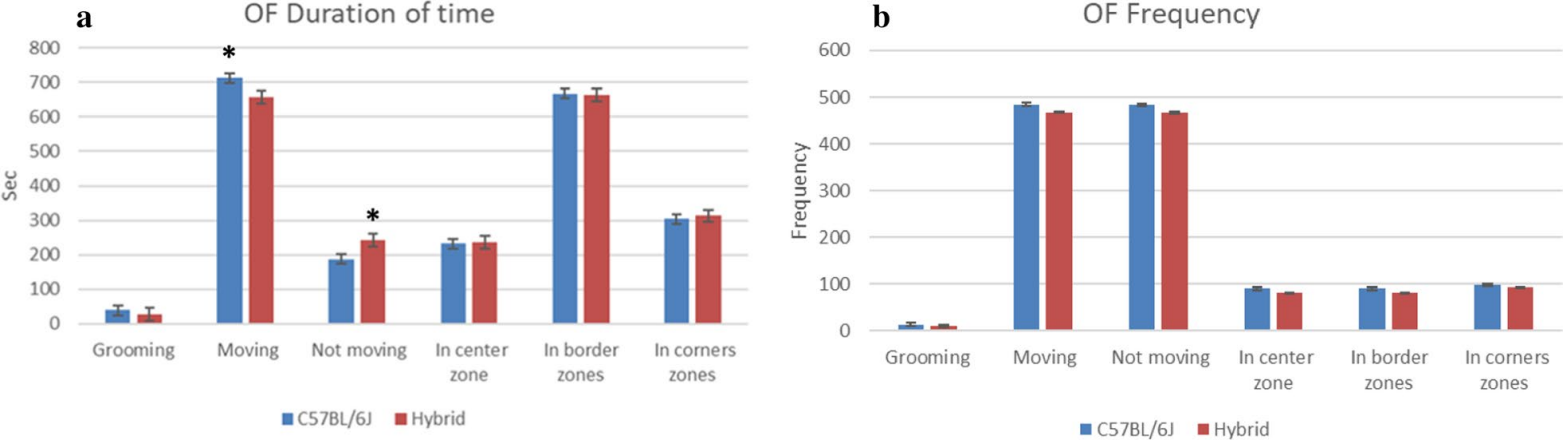
Summary of the results from the open field test of two different mouse strains (C57BL/6J and hybrid B6129SF1/J). Results are presented as means (± SE) and indicated with * when significant (p ≤ 0.05). Total distance moved is not included. **a** shows the duration of time spent grooming, moving, not moving and in the different zones, presented in seconds. **b** shows the frequency of grooming, moving, not moving and in the different zones

The C57BL/6J were moving more and covering more ground during the OF than the hybrid, but only the time spent moving was significantly different from the hybrid (p = 0.025). Consequently, the time spent not moving was also significantly different between the strains (p = 0.025, Fig. 1a). The number of fecal pellets left by the hybrid was significantly higher (p < 0.004) than by the C57BL/6J, and also, the number of urine puddles had the same trend for the hybrid, but was not significantly higher (Table 1).

**Table 1:** Results open field and fecal pellets/urine puddles

Strain	Mean distance moved in total (cm)	Mean time spent moving (s)	Mean time spent not moving (s)	Mean time spent in center zone (s)	Mean time spent in corner zones (s)	Mean number of fecal pellets OF	Mean number of urine puddles OF	Mean number of fecal pellets BM	Mean number of urine puddles BM
C57BL/6J	7088	**713**	187	233	304	0.30	0.20	0.33	0.08
Hybrid B6129SF1/J	6239	657	**243**	237	313	**0.90**	0.40	**0.70**	0.20

Summary of results from open field test and fecal pellets/urine puddles (left in open field and Barnes maze) from two different mouse strains (C57BL/6J and hybrid B6129SF1/J). Results are presented as means. Differences between the strains were assessed with Student’s t-test and indicated in bold when significant (p ≤ 0.05)

### Barnes maze (BM) test

**Fig. 2:**
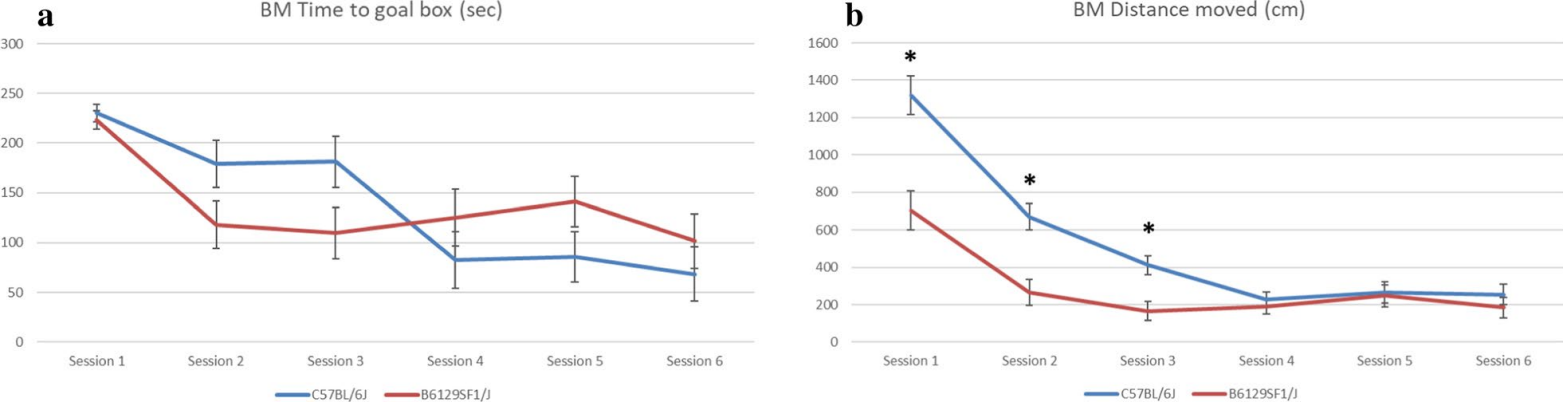
Summary of the results from the Barnes maze test of two different mouse strains (C57BL/6J and hybrid B6129SF1/J). Results are presented as means (± SE) and indicated with * when significant (p ≤ 0.05). **a** shows time spent to find the goal box, presented in seconds. **b** shows distance moved before the mouse entered the goal box, presented in centimetres

Both mouse strains showed progress in learning as the time used before entering the goal box decreased for each session, except for the fourth and fifth sessions for the hybrid and fifth session for C57BL/6J (Fig. 2a). The hybrid was slightly, but not significantly quicker than C57BL/6J in sessions two and three. In the three last sessions, the C57BL/6J was slightly quicker, also not significantly. The hybrids covered significantly less ground before they reached the goal box in the three first sessions, than the C57BL/6J (p < 0.001, p < 0.001, and p = 0.003, respectively) (Fig. 2b). The number of fecal pellets left by the hybrid was significantly higher (p < 0.001) than by the C57BL/6J, and also, the number of urine puddles had the same trend for the hybrid, however this was not significant (Table 1).

### Stress test

**Fig. 3:**
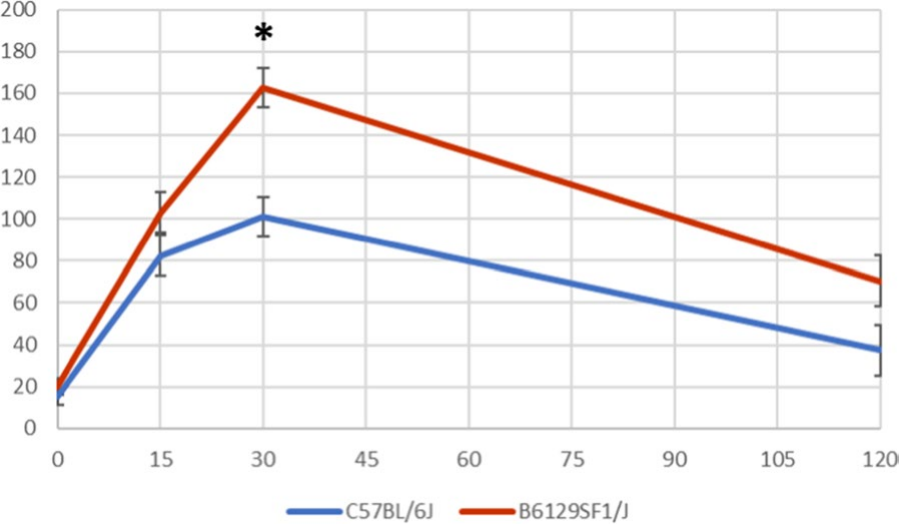
Summary of the results from the stress test of two different mouse strains (C57BL/6J and hybrid B6129SF1/J). Results are presented as means (± SE) of blood concentration of corticosterone (ng/ml) at each different timepoint (minutes) and indicated with * when significant (p ≤ 0.05)

Both strains had a similar baseline of corticosteroid blood levels at the starting point of the stress test. The C57BL/6J did have a slightly lower concentration of corticosterone at all time points after that, resulting in the response curve of the C57BL/6J being flatter than the hybrid’s (Fig. 3). However, the only significant effect of mouse strain was at 30 min (p < 0.001) which was the time-point of peak concentration in both strains.

## Discussion

In the present study, we compared two different types of mouse strains (C57BL/6J and hybrid B6129SF1/J) using three different behavior tests (OF, BM and stress test). We found that the C57BL/6J were more locomotory active during the OF by spending more time moving than the hybrid. Both strains learned the BM, but the hybrid covered less ground before they found the goal box during sessions 1, 2 and 3. During the stress test, the C57BL/6J had a lower stress-induced corticosterone response than the hybrid.

As this small scale study only includes young males, more studies should be done to fully understand the differences between these two mouse strains. As mentioned, the reason for choosing the sex and age of the animals was done to avoid sexual behavior and female reproductive cyclic variation disturbing the results. The mice were single housed from the day prior to test start, to avoid fighting during the testing period. This change in housing close to test-start is not optimal as it can cause distress that might affect the results. On the other hand, the alternative, group- or pair-housing, could lead to fighting and building of hierarchies that would also affect the results. Nevertheless, the change should ideally have been performed with more time to acclimatization to the facility and to the new situation of being single-housed.

### Open field (OF) test

The OF measures locomotor activity and anxiety-like behavior in mice [6]. Under normal conditions, mice will do explorative behavior and use the whole box and also venture out into the center zone of the box. Mice with anxiety will spend more time in the corners, trying to find a place to hide. As these behavioral differences between groups of mice can be confounded by differences in exploratory and/or locomotor activity, it is suggested that more tests should be performed in order to conclude from an OF study. Furthermore, a good alternative for measuring locomotor activity would be to track the mice in their home cage during the night, when the mice are active [7].

In the present study, both mouse strains spent the same amount of time in both the center zone, the border zones and the corner zones, but the C57BL/6J did spend significantly more time moving, suggesting this mouse strain to be more explorative and active. Although there was a trend, distance moved in the OF was not different between the two strains. Other studies also found a higher activity level of the C57BL/6J than the hybrid in the OF. Bolivar et al*.* compared inbred strains and F1 hybrids of 129S3/SvImJ, A/J, BALB/ cByJ, C3H/HeJ, CBA/J, DBA/2J, FVB/NJ, (B6 × 129)F1/J and (B6 × C3H) F1/J. This study confirmed that the genetic differences in the different strains affect the intersession habituation to the OF. In their results they ranked the strains by total distance traveled in the OF and the C57BL/6J ranked higher than the B6129F1 hybrid, for both males and females [3]. Logue et al*.* also compared twelve different strains of inbred mice and seven F1 hybrids in several behavioral tests including OF, and they also found the C57BL/6J to be more active, although they used a 129B6F1 hybrid [8].

The lack of strain differences in time spent in the center zone indicates no strain differences in anxiety, however, a significantly higher frequency of defecation by the hybrid could suggest this strain to be more anxious. The release of urine and feces is the reaction to the fight-or-flight response that comes with the stress response, in many species [9], also mice. Originally, OF was designed to record defecation as a measure of emotionality [7].

### Barnes maze (BM) test

There are several different versions of BM tests and protocols. The protocol we chose has been validated and frequently used for learning performance [10, 11]. The BM measures spatial learning and memory in mice [12]. It is an alternative to the Morris Water maze and offers the advantage of being free from the potentially confounding influence of swimming behavior. The BM will measure learning impairments, as shown by a study in which induced traumatic brain injury in C57BL/6 mice resulted in a significantly longer time to learn the BM [13]. Under normal conditions, mice will use the spatial cues to remember where their goal box is, and adding motivators to succeeding sessions will motivate them to perform quicker for each session. O’Leary and Brown tested the C57Bl/6J in different BM scenarios and found that mice do not use the visuospatial cues to locate the escape hole on the small-diameter maze (69 cm) with a wall and intra-maze visual cues, but they do use the visuospatial cues on small or large diameter mazes (122 cm) with no wall [14]. In a different study, this research group tested 13 inbred strains in the BM, and found that the use of visuospatial cues is dependent on the strain and their visual ability, as some stains have reduced sight [5]. There is a theory, put forth by Illouz et al*.*, that mice use different strategies to find the goal box, some use the spatial cues actively and go straight for the goal box, and others randomly search the maze until they find it, using speed as their strategy [15]. The fact that the hybrid of the current study covered less ground to find the goal box during sessions 1, 2 and 3 may again indicate that this strain is less explorative and active than C57BL/6, however, it may also mean they used different search strategies. A cued strategy will require less ground than a serial or random strategy. Some mice have a strong preference for using the room cues in their strategy [16]. Still, the hybrid was not significantly faster than C57BL/6 in finding the goal box, therefore, C57BL/6J more likely used speed and exploration in its search. O’leary et al*.* also discuss that mice from the 129 sub-strains show lower levels of exploration than the C57BL/6. This fits well with our observations of the hybrid containing this strain.

In the present study, motivators were used to avoid habituation of the repeated session. In the first two sessions, the motivator was a bright light, and in the third and fourth sessions, a fan was added. In the fifth session, the third motivator, a buzzer, was added. The motivators used in the current study seemed to affect the hybrid more severely than C57BL/6 as their progress was reversed during sessions 4 and 5, while the progress of C57BL/6 was only slightly reversed during session 5. Using stressful motivators can combine testing of learning and stress handling [17], and the motivators may distract the mice in their learning [18]. In this study, the motivator added in session 5 seemed to distract both strains and did not serve its purpose. Using positive motivators could be a solution if motivation is needed. Youn et al*.* showed that DBA/2J mice, that originally performed poorer than the C57BL6J mice in the BM, first trained with no motivators and then with a fan as a motivator, actually outperformed them when including a positive motivator (almond chips) [19].

### Stress test

There are many different tests measuring stress response in mice, for example, the tail suspension test and chronic restraint stress tests. Choosing the correct test would have to be a compromise between good animal welfare and comprehensive results. Taking these into account, we chose the test restraining the mice in falcon tubes for 15 min [20]. This test is often called a hypothalamus–pituitary–adrenal axis responsivity test, as it indicates a reaction in this endocrine axis. As mice are prey animals, being restrained in a transparent tube, without the ability to flee or hide, naturally creates a stress response. After the 15 min restraint, the mouse is allowed to calm down in its home cage, and the levels of corticosterone will slowly go back to normal levels. This stress hormone response is critical for the survival of all species, but chronic high levels of stress hormones have adverse effects on health [21] and may result in mental problems [22, 23]. Therefore, the best way for the body to handle stress is to elicit an appropriate stress hormone response to a stressor followed by efficiently lowering the levels when the threat subsides.

In the present study, the baseline concentration was almost similar for the two strains. Expectedly, both mouse strains responded with elevated corticosterone concentrations following the restraint. However, the hybrid had significantly higher levels after 30 min than the C57BL/6J. This shows that the C57BL/6J has a lower corticosterone response to stress, while the hybrid, reacts more severely in terms of corticosterone response to the same acute stressor. Chan et al*.* also performed a similar study. They used the stress test to compare the C57BL/6, the 129, and the C57BL/6:129 hybrids. The F1 hybrid (BL/6 mother and 129 father) showed similar corticosterone levels (150 ng/ml), as observed in the current study (162.8 ng/ml), however, interestingly, the C57BL/6 mice had the highest stress-responsive levels. It rose to over 200 ng/ml after 15 min of restraint [4].

## Conclusions

Taken together, the adolescent males of the two strains compared behaved differently in the behavior tests and reacted differently to restraint stress in terms of corticosterone levels. C57BL6/J spent more time moving in the OF and moved a longer distance to reach the goal box in the three first sessions of the BM than the hybrid. This may indicate a higher locomotor activity and/or explorative behavior in C57BL6/J, although search strategy in the BM also may play a role. This presumably more physically active, explorative strain reacted with a lower corticosterone increase than the hybrid to restraint stress. The hybrid appears more sensitive to stressors as they left more feces in both the OF and the BM, and showed a higher corticosterone response to restraint. If only performance in these behavior tests were taken into account, the C57BL6/J strain would be the most robust. However, there might be many other traits of interest when choosing a model strain. Again, it must be emphasized that these results are only valid for adolescent males of the two strains as no females or mice at other ages were tested.

## Methods

### Ethics statement

The study was performed at the Section for Experimental Biomedicine at The Norwegian University of Life Sciences in Oslo, Norway. The animal facility is licensed by the Norwegian Food Safety Authority (https://www.mattilsynet.no/language/english/) and accredited by the Association for Assessment and Accreditation of Laboratory Animal Care (https://www.aaalac.org/). The animal experiment was approved by the unit’s animal ethics committee (Institutional Animal Care and Use Committee/IACUC) and the Food Safety Authority (application ID: FOTS 4247, 2013/39783) and executed in compliance with the local and national regulations associated with laboratory animal experiments. The rodent and rabbit section of the facility is a Specific Pathogen Free (SPF) unit. It follows a health monitoring program recommended by the Federation of European Laboratory Animal Science Associations/FELASA (http://www.felasa.eu/). The care of the animals was carried out by two veterinary nurses with FELASA B certification and three researchers with FELASA C certification performed the experiments.

### Animal models

10 male C57BL/6 J mice and 10 male hybrid B6129SF1/J mice (both from Jackson Laboratory, Maine, USA) were used. The mice were 5 weeks old at arrival and were acclimated to the unit for one week before testing started. The age and sex of animals were chosen to avoid female reproductive cyclic variation and as males tend to track the females in behavior tests, which could interfere with results in such a small scale study.

### Housing and husbandry

The animals were housed in open type III cages (Tecniplast, Buguggiate, Italy) in groups of 5 during the acclimatization time and single housed from the day prior to the first test and during the testing period. The reason for the single housing during the testing period was to avoid stress due to fighting during the testing. The cages contained standard aspen bedding (Scanbur BK, Nittedal, Norway), cellulose nesting material and a Bio-Serv igloo as a hide (Bio-Serv, Frenchtown, NJ, USA). The animals were given a standard maintenance diet (RM1 from SDS, Witham, UK) and tap water ad libitum. The animal room was on a 12:12 light–dark cycle, from 08.00 in the morning and 20.00 in the evening, with a room temperature of 21 ± 2 ^0^C with 20 air changes per hour and 45 ± 5% relative humidity. The cages and bedding were changed twice a week for group-housed mice and once a week for single housed mice, and the water was changed daily. The animals were not disturbed 24 h before testing.

All mice were tested in all 3 behavior tests: First OF test on day 1, then BM test on day 2–4 and then the stress test on day 5. The OF and BM were performed in a procedure room next to the housing room, while the stress test and the following blood sampling took place inside the housing room, to avoid transport stress just prior to the test. After behavioral testing, all animals were euthanized using cervical dislocation.

### Open field (OF) test

The OF testing was done in the animals` light cycle, after working hours (16.00–20.00), for calm testing conditions. The OF testing arena was a white plexiglass box 50 × 50 × 22 cm (Noldus, Wageningen, the Netherlands) with a bright light (Lupoled 1120; approx. 120 lx) placed above. The animal was lifted by the tail, carried on the arm and gently placed inside a disposable nontransparent cardboard cylinder (guinea pig play tunnel from Scanbur BK, Nittedal, Norway) in the center of the arena and left there for 3 s. When released from the cylinder, each mouse was tracked for 15 min. The animals were filmed by an Ikegami ICD-49E B/W infrared camera fixed in the ceiling and tracking was done by the computer program Ethovision XT 9.0 (Noldus, Wageningen, the Netherlands). Urine puddles and fecal pellets left in the arena were recorded manually. During the analysis, the floor in the box was divided into 3 zones: Center zone, corner zones and border zones alongside the walls of the box. The three researchers performing the experiments had fixed tasks, one handling the animals, one performing the tracking and one preparing the testing arena between each test.

### Barnes Maze (BM) test

The BM testing was done in the animals` light cycle during working hours (08.00–16.00). The BM testing arena was a round platform, 100 cm in diameter, with 20 holes, one of which had a black goal box beneath (Noldus, Wageningen, the Netherlands). There were spatial room cues placed on the walls, to help the mice navigate. The animal was handled the same way and filmed by the same camera and tracking was done by the same computer program, as for OF. Each mouse was tracked until it reached the goal box or for 4 min. If the animal had not located the goal box by 4 min, it was gently guided to the box. All animals were trained for 2 sessions every day, morning and afternoon with four hours in between the sessions, for 3 days. To account for the risk of reduced motivation that occurs in repeated tasks a new motivator was added every day as described by Müller and Bale [10]:

- Day 1: Session 1 + 2: Bright light over the platform (Lupoled 1120; approx. 120 lx).
- Day 2: Session 3 + 4: Bright light + blowing fan.
- Day 3: Session 5 + 6: Bright light + blowing fan + high sounding buzzer.

Urine puddles and fecal pellets left on the platform were recorded manually. The three researchers performing the experiments had fixed tasks, one handling the animals, one performing the tracking and one preparing the testing arena between each test.

### Stress test

The stress test was performed in the animals’ light cycle during working hours (08.00–16.00). The mouse was restrained inside a 50 mL falcon tube for 15 min, on a table next to the home cage. Blood samples were taken from the tip of the tail at 4 different time points: 0 (start), 15 (before release), 30 and 120 min (after release). The mouse was allowed to rest in its home cage in between the 15, 30 and 120 min samples. All samples were taken with a 20 µL Minivette POCT capillary collecting tube coated with EDTA (Sarstedt, Nümbrecht, Germany) and transferred to an Eppendorf tube on ice. The blood samples were spun at 5000 rpm at 4 °C for 10 min to obtain the plasma, which was stored at − 80 °C until further analyses. Corticosterone was measured in the plasma using an MP Biomedicals ImmuChem™ Double Antibody Corticosterone 125 Ria Kit (MP Biomedicals, Santa Ana, CA, USA) according to the manufacturer’s instructions.

### Statistical analyses

The results of the data were analyzed in JMP Pro 13 ® (SAS, Cary, NC, USA) comparing the two groups using the Student’s t-test. Corticosterone levels were analyzed across time, using a Student’s t-test for each time Bar graphs and line charts were made in Excel ® (Microsoft, USA) presenting the data in means. We chose not to include the data from the total distance moved in the OF figure, not to misalign the axis. P-values ≤ 0.05 were considered statistically significant.

## Data Availability

All data is submitted as tables and figures in PDFs included in the manuscript.

## Abbreviations

BM: Barnes maze test
FELASA: Federation of European Laboratory Animal Science Associations
IACUC: Institutional Animal Care and Use Committee
OF: Open field test
SPF: Specific Pathogen Free
